# Eddy Current Classification of Sheet Ferromagnetic Materials Based on Material Property Profiles

**DOI:** 10.3390/s26154967

**Published:** 2026-08-05

**Authors:** Volodymyr Ya. Halchenko, Ruslana Trembovetska, Volodymyr Tychkov, Viacheslav Kovtun, Oles Stankevych

**Affiliations:** 1 Department of Instrumentation, Mechatronics and Computerized Technologies, Cherkasy State Technological University, 460 Shevchenko Blvd., 18006 Cherkasy, Ukraine; v.halchenko@chdtu.edu.ua (V.Y.H.); r.trembovetska@chdtu.edu.ua (R.T.); v.tychkov@chdtu.edu.ua (V.T.); o.s.stankevych.asp25@chdtu.edu.ua (O.S.); 2 Department of Computer Control Systems, Faculty of Intelligent Information Technologies and Automation, Vinnytsia National Technical University, Khmelnitske Shose Str., 95, 21000 Vinnytsia, Ukraine

**Keywords:** fine-grade material sorting, suppression of the effects of uncontrollable factors, GAN inversion, latent space, Taguchi optimization

## Abstract

This paper proposes a novel strategy for the fine-grade classification of sheet ferromagnetic materials with similar electromagnetic properties. The strategy integrates three original components: suppression of the effects of uncontrollable factors on the probe signal, reconstruction of material property profiles, and classification in a reduced-dimensional latent space. Together, these components constitute an innovative framework for effectively identifying sheet samples using the eddy current method. The strategy can be summarized as follows: interference-suppressed eddy current probe signal → GAN latent space → distance-to-class classifier → material grade. This part of the study focuses on generalizing, extending, and adapting the method previously developed by the authors for acquiring signals directly from a Taguchi-optimized probe without prior signal processing. The method is applied to the indirect measurement of material property profiles, which constitutes the first stage and a key prerequisite for implementing the proposed sample-identification concept. The results of the numerical experiments demonstrate that the optimally designed probe provides a high signal-to-noise ratio and an acceptable reduction in output-signal variations caused by uncontrollable factors.

## 1. Introduction

Metal grade verification and product classification by grade are common industrial tasks that require reliable non-destructive material identification methods. One of the most effective and widely used techniques in this context is eddy current testing [1,2,3,4,5,6], which meets current industrial requirements and offers distinct advantages over alternative methods. Steel grades with similar properties are difficult to classify accurately because multiple factors can distort the measured response and lead to misclassification. Class overlap in the feature space is therefore common and requires specialized signal processing and data analysis methods to improve class reparability and reduce misclassification probability. 

Material-grade classification requires reference samples whose properties are representative of each grade. The tested object is then compared with these references and assigned to the corresponding class. The parameters used to identify a material grade typically include its electrical conductivity, magnetic permeability, and chemical composition. It is therefore appropriate to regard the grade of a material as the totality of its characteristics, as revealed by its response to an electromagnetic field. These characteristics serve as the features used to identify the material. These features must be relatively easy to obtain while remaining sufficiently informative to distinguish reliably among different material classes. Accordingly, recent feature-selection approaches tend to use more complex but more informative features.

The amplitude and phase characteristics of probe signals are commonly used to classify materials. For example, Qian et al. [7] proposed a low-frequency differential eddy current detection method for identifying and classifying raw materials for the manufacture of steel bearing bars. Their approach involved numerical simulations of the eddy current density distribution within the bars and of the effects of the excitation signals on the signals induced in the probe. Subsequently, an experimental system for detecting low-frequency eddy currents using a phase-locked amplifier algorithm was developed A similar material-classification technique was presented in [8], in which an eddy current method was proposed for characterizing and classifying different aging states of heat-resistant austenitic steel tubes. To characterize the samples, the amplitude and phase shift of the eddy current signals are calculated and used as features. A machine learning algorithm was then used to classify the test samples on the basis of the extracted features. However, the methods considered do not reduce the effects of signal noise caused by uncontrollable interference to an acceptable level.

In many cases, the grade of a material can be determined by measuring its electrical conductivity indirectly, i.e., by inversely extracting the relevant information from the probe signal [9], which depends on alloying additions and microstructural changes induced by processing or other factors. However, this classification criterion is more commonly applied to nonmagnetic materials, such as aluminum and titanium alloys. 

If the tested object is ferromagnetic, additional errors arise in conductivity measurements because *σ* and *μ* simultaneously affect the probe signal. Moreover, informational uncertainty often arises because this feature is not unique: the electrical conductivity ranges of different material grades may overlap. Even materials of the same grade produced by the same manufacturer may exhibit electrical conductivity variations of approximately ±5–7% relative to the mean because of numerous influencing factors.

The eddy current method is also suitable for classifying ferromagnetic materials with similar properties, particularly carbon and low-alloy steels, although their classification is more complex than that of nonmagnetic materials. Thus, magnetic materials can be classified using an inversely determined feature, namely magnetic permeability, as demonstrated in [10]. However, this approach has the same limitations as the previous one.

Each steel grade has a characteristic electromagnetic signature resulting from its electrical conductivity and magnetic permeability. In single-frequency identification, the two parameters, *σ* and *μ*, can be obtained from a single measurement using an *σ*–*μ* meter [11]. However, the applicability of this approach is limited by the strong interaction between *σ* and *μ* and the difficulty of separating their effects, as demonstrated in the cited study. Nevertheless, current research shows a trend toward increasing the information content of classification features.

Classification features were further developed through the use of multi-frequency eddy current testing (MECT). MECT provides richer information about electromagnetic signatures by measuring the material response at multiple frequencies. In addition to conventional MECT, pulsed eddy current testing (PECT) has become widely used for material characterization. In addition to potentially increasing the number of classification features available for material sorting, both eddy current excitation modes provide effective means of suppressing measurement interference caused by factors such as lift-off and local variations in object geometry. If these effects are neglected, reliable material identification becomes impossible. Qian et al. [12] proposed a PECT-based approach that combines multidimensional time- and frequency-domain features with a neural network classification algorithm for steel grade recognition. Qian et al. [13] proposed a dynamic nondestructive testing method based on differential pulsed eddy currents and a convolutional neural network that adaptively combines dual-branch spatiotemporal features with statistical signal features. The method enables both the classification of known classes and the recognition of unknown classes in moving bearing-steel products. This group of material-sorting methods also includes magnetic induction spectroscopy (MIS), which analyzes a material’s response to a variable magnetic field over a broad frequency range. By varying the excitation frequency, MIS provides multi-parameter information associated with different penetration depths in the tested object. Thus, there is a clear trend toward increasing the information content of classification features. Studies [14,15,16] investigated the application of this method to nonmagnetic materials. A major limitation of these methods is the increased hardware complexity of the eddy current measurement system. Ideally, the same advantages should be achieved using a single-frequency configuration.

These limitations motivate alternative single-frequency approaches to material sorting by grade. A further development of this approach involves using material property profiles as classification features, specifically, the distributions of *σ* and *μ* as functions of electromagnetic field penetration depth in the tested object. These distributions can be obtained by measuring the probe signal at a single fixed frequency and applying numerical inversion techniques.

Studies [17,18,19] describe the indirect measurement of material property profiles using surface transformer probes. The numerical inversion technique employed in these methods is based on surrogate optimization using neural network proxy models [20]. However, the characteristics of the exact Uzal–Cheng–Dodd–Deeds magnetodynamic model result in a large number of optimization variables. These variables correspond to points on the continuous *σ* and *μ* profiles represented using a piecewise-constant approximation, thereby reducing the accuracy with which the optimum can be determined. The transition to lower-dimensional spaces using the PCA and kernel PCA machine-learning methods in [21,22] did not yield a satisfactory improvement in the results. This outcome was apparently caused not only by the relatively high dimensionality of the profile search space but also by the limited generalization capability of surrogate deep-learning models trained on a discrete dataset and intended to generate candidate profiles in a continuous domain. Adequately addressing these two issues is necessary to solve the problem of simultaneously determining the inversion parameters, that is, the *σ* and *μ* profiles, with acceptable accuracy. These problems could be addressed simultaneously by using generative artificial intelligence models, particularly generative adversarial networks (GANs), to solve the inverse electrodynamic problem. This choice is justified because GANs are capable of learning the complex interaction between *σ* and *μ*. GANs learn the manifold of physically plausible profiles across the complete set of metal grades. A pre-trained GAN is used to generate a continuous synthetic representation of physically plausible material property profiles in a high-dimensional data space. This process is controlled by a code associated with each profile and drawn from a latent space whose dimensionality is substantially lower than that of the data space. The latent space is an abstract parametric space in which the GAN encodes a priori information about physically permissible profiles of the tested object’s properties. In this context, information content is represented at the distributional level. This capability allows the subsequent calculations to be performed in a continuous latent space of the lowest practicable dimensionality. The application of regularization techniques in GANs constrains the reconstructed profiles, thereby ensuring their physical plausibility.

Additionally, this approach makes it possible to perform the classification stage of the sorting procedure not in a conventional high-dimensional data space but in the substantially lower-dimensional latent space of a generative model, where each metal grade forms a compact, structured region. In the latent space, conductivity and magnetic permeability profiles can be compared without explicit normalization, despite their different physical units, value ranges, and variability scales.

However, reliable classification of magnetic materials by grade is practically impossible unless the effects of uncontrollable interference factors on eddy current probe measurements are suppressed. For this purpose, the method developed by the authors can suppress these effects without requiring the physical elimination of their sources. As reported in [23,24,25,26], the method has been applied to numerous eddy current measurement problems, including the determination of electrical conductivity, magnetic permeability, metal plate thickness, and the thicknesses of dielectric and metallic coatings on nonmagnetic and ferromagnetic substrates. It has also been used to measure geometric anomalies and increase the signal-to-noise ratio, demonstrating both effectiveness and high application specificity. It does not involve the use of algorithms for processing the selected signals. Interference-suppressed signals are obtained directly from the probes through the robust parametric design of Taguchi-optimized configurations with reduced sensitivity to interference. The method can be generalized and adapted to surface probes used for magnetic metal grade classification while accounting for the relevant measurement interference factors.

Therefore, to summarize, there is currently a need for a systematic solution to the problem of the fine-grade classification of ferromagnetic metals, which is linked, in particular, to providing more comprehensive information regarding the classification features, which are characterized by the ambiguity of a significantly non-linear mapping between material parameters and the signal measured by the probe, their differing physical nature, the suppression of the influence of interfering external factors and, consequently, additional signal variations, which are not determined by class membership, as well as the absence of adequate integrated metrics that account for the correlations between heterogeneous features and their varying sensitivity, that is, metrics consistent with the physics of the signal formation process.

This study formulates a new framework for the reliable fine-grade classification of sheet ferromagnetic materials when the properties of different grades are highly similar. In such cases, the classification features have limited discriminatory power because different physical conditions may produce virtually indistinguishable probe signals. Schematically, the classification strategy is as follows: interference-suppressed eddy current probe signal → GAN latent space (GAN inversion) → distance-to-class classifier → material grade. In the latent space of the generative model, the representations corresponding to different steel grades form compact, spatially separated clusters. The structure of each cluster reflects the variability in electromagnetic properties within the corresponding material grade, whereas the distances between clusters indicate the degree of separability among the grades. Accordingly, a novel element of the proposed classification framework is the use of inverse features, that is, material-property profiles obtained by solving the inverse problem from single-frequency measurements of the probe signal amplitude and phase. This approach further improves the information content of classification features used in this field without increasing hardware or software complexity.

The novelty of this study lies in the integration of three original methods: interference suppression, material property profile reconstruction, and latent space classification. Together, these methods constitute a new framework for eddy current classification.

The aim of the presented research is to describe the general principles of the proposed methodology for classifying materials, one of the main stages of which is the generalization, development, and adaptation of the method developed by the authors for suppressing the influence of noise factors on the profile gauge output signal of the object’s material properties. The method identifies eddy current probe designs that maximize the signal-to-noise ratio in accordance with Taguchi’s methodology and does not require additional signal processing. Robust probe design constitutes the first indispensable and functionally integrated stage of the proposed methodology. It generates measurement signals with an enhanced signal-to-noise ratio, thereby providing the conditions necessary for robust inverse-feature identification and reliable material classification.

## 2. Materials and Methods

### 2.1. Design of an Eddy Current Profile Gauge

In sorting sheet ferromagnetic materials on the basis of their electrophysical property profiles, the design of the surface eddy current probe (SECP) used as a profile gauge plays a crucial role.

**Figure 1:**
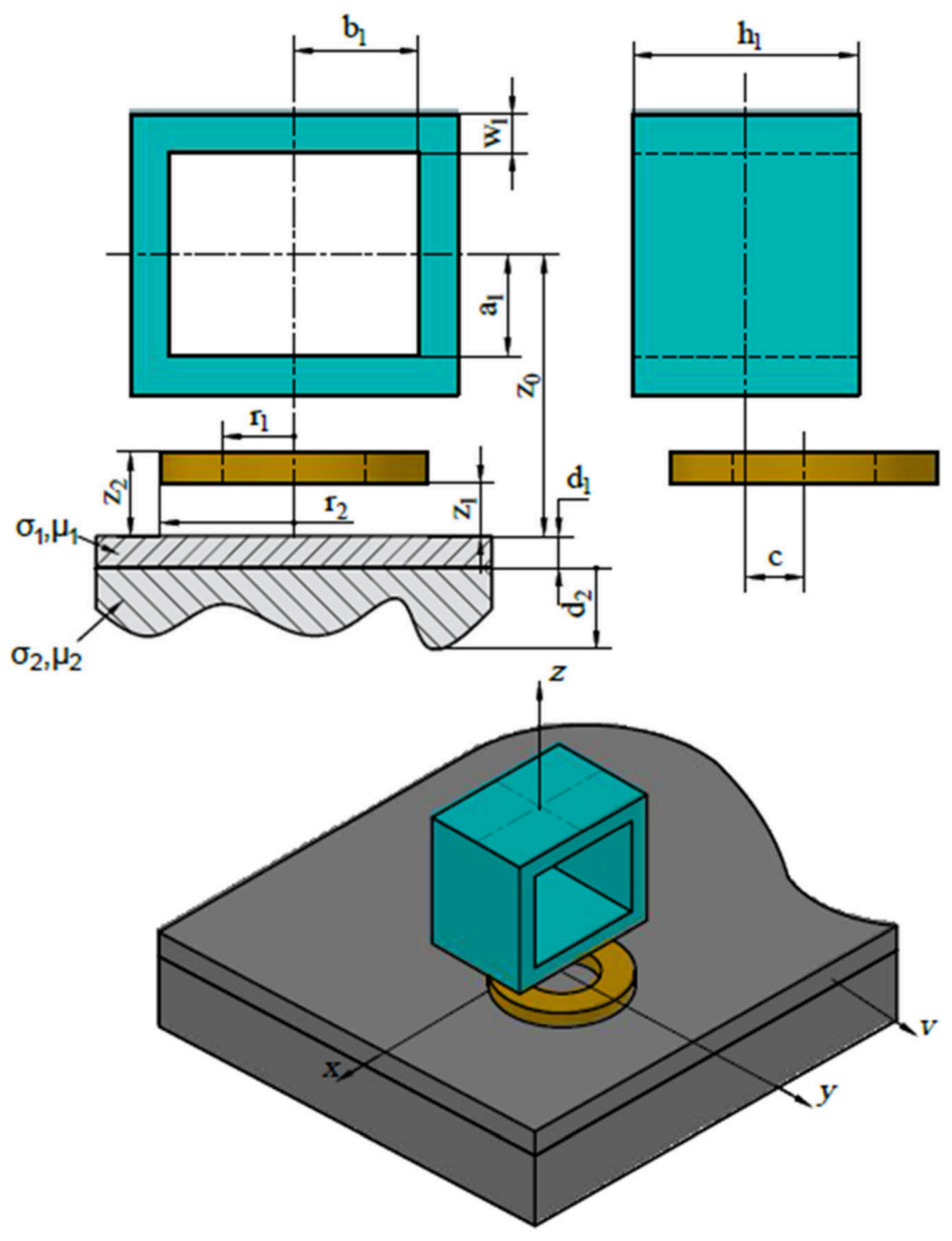
Tangential probe with a pancake-shaped circular pick-up coil.

Recent studies have shown considerable interest in self-differential and self-nulling transformer configurations of SECPs [27,28,29,30]. Halchenko et al. [24] investigated an SECP with these characteristics for measuring the electrical conductivity of a substrate beneath a conductive coating on a moving tested object (TO). The probe demonstrated promising performance in suppressing the effects of uncontrollable factors. Its application to the indirect measurement of electrical conductivity and magnetic permeability profiles required for material sorting is therefore a logical extension of the method. The design consists of a rectangular excitation coil positioned perpendicular to the TO surface and a cylindrical pickup coil oriented parallel to it. Figure 1 schematically illustrates the design of the tangential self-differential SECP and its position relative to the TO. The figure depicts a moving two-layer TO; however, in the present study, the TO is modeled as a stationary sheet composed of a single homogeneous ferromagnetic material. This condition is imposed in the mathematical model in Equation (1) by setting the variables as follows: *µ*_1_ = *µ*, *µ*_2_ = 1, *σ*_1_ = *σ*, *σ*_2_ = 0, *d*_1_ = *d*, *d*_2_ = ∞, and *v* = 0.

Assuming that the TO medium is linear and isotropic, the mathematical model used to calculate the EMF induced in the probe by a sinusoidal excitation current $Ie^{j\omega t}$ with angular frequency *ω* is expressed as follows [30]:

(1) $$\begin{array}{c} E_{c}=\frac{-\omega \mu_{0}IN_{1}N_{2}}{\pi w_{1}h_{1}\left(r_{2}-r_{1}\right)\left(z_{2}-z_{1}\right)}\int_{-\infty }^{\infty }\int_{-\infty }^{\infty }\frac{\kappa {\hat{k}}_{1}}{\xi \zeta^{5}}sin\left(\frac{\eta h_{1}}{2}\right)e^{-j\eta c}\times Int\left(r_{1}\zeta \;,\;r_{2}\zeta \right)\\ \times \left[e^{-\zeta \left(z_{0}+z_{1}\right)}-e^{-\zeta \left(z_{0}+z_{2}\right)}\right]d\xi d\eta \end{array}$$

where

(2) $$Int\left(r_{1}\zeta ,\;r_{2}\zeta \right)=\int_{r_{1}\zeta }^{r_{2}\zeta }xJ_{1}\left(x\right)dx$$

(3) $$\kappa =\frac{\left(\frac{\gamma_{1}}{\varsigma }-\frac{\mu_{1}}{\mu_{0}}\right)\times \left(\frac{\gamma_{2}}{\gamma_{1}}+\frac{\mu_{2}}{\mu_{1}}\right)+\left(\frac{\gamma_{1}}{\varsigma }+\frac{\mu_{1}}{\mu_{0}}\right)\times \left(\frac{\gamma_{2}}{\gamma_{1}}-\frac{\mu_{2}}{\mu_{1}}\right)\times e^{-2\gamma_{1}d_{1}}}{\left(\frac{\gamma_{1}}{\varsigma }+\frac{\mu_{1}}{\mu_{0}}\right)\times \left(\frac{\gamma_{2}}{\gamma_{1}}+\frac{\mu_{2}}{\mu_{1}}\right)+\left(\frac{\gamma_{1}}{\varsigma }-\frac{\mu_{1}}{\mu_{0}}\right)\times \left(\frac{\gamma_{2}}{\gamma_{1}}-\frac{\mu_{2}}{\mu_{1}}\right)\times e^{-2\gamma_{1}d_{1}}}$$

(4) $$\begin{array}{l} {\hat{k}}_{1}=\left[\frac{\zeta sin\xi \left(b_{1}+w_{1}\right)-\xi cos\xi \left(b_{1}+w_{1}\right)}{\xi^{2}+\zeta^{2}}\right]e^{\zeta \left(a_{1}+w_{1}\right)}-\left[\frac{\zeta sin\xi b_{1}-\xi cos\xi b_{1}}{\xi^{2}+\zeta^{2}}\right]e^{\zeta a_{1}}+\\ +\left[\frac{\zeta sin\xi \left(b_{1}+w_{1}\right)+\xi cos\xi \left(b_{1}+w_{1}\right)}{\xi^{2}+\zeta^{2}}\right]e^{-\zeta \left(a_{1}+w_{1}\right)}-\left[\frac{\zeta sin\xi b_{1}+\xi cos\xi b_{1}}{\xi^{2}+\zeta^{2}}\right]e^{-\zeta a_{1}} \end{array}$$

(5) $$\gamma_{1}=\sqrt{\xi^{2}+\eta^{2}-j\sigma_{1}\mu_{1}\nu \eta +j\omega \sigma_{1}\mu_{1}}$$

(6) $$\gamma_{2}=\sqrt{\xi^{2}+\eta^{2}-j\sigma_{2}\mu_{2}\nu \eta +j\omega \sigma_{2}\mu_{2}}$$

(7) $$\varsigma =\sqrt{\xi^{2}+\eta^{2}}$$

where *ξ* and *η* are Fourier transform integration variables, *µ*_0_ = 4·π·10^−7^ (H/m) is the magnetic constant in vacuum, *d*_1_ (m) is the coating thickness, *a*_1_ (m) is the width of the excitation coil, *b*_1_ (m) is the length of the excitation coil, *h*_1_ (m) is the height of the excitation coil cross-section, *c* (m) is the center-to-center distance between the pick-up and excitation coils, *w*_1_ (m) is the width of the excitation coil cross-section, *N*_1_ is the number of turns in the excitation coil, *N*_2_ is the number of turns in the pick-up coil, *µ*_1_ (H/m) is the absolute magnetic permeability of the coating, *µ*_2_ (H/m) is the absolute magnetic permeability of the substrate, *σ*_1_ (S/m) is the electrical conductivity of the coating, *σ*_2_ (S/m) is the electrical conductivity of the substrate, *v* (m/s) is the speed, *J*_1_(*x*) is the cylindrical Bessel function of the first kind and first order, *r*_1_ (m) is the inner radius of the cylindrical pick-up coil, *r*_2_ (m) is the outer radius of the cylindrical pick-up coil, *z*_1_ (m) is the lift-off, *z*_2_ (m) is the distance between TO and the upper edge of the cylindrical pick-up coil, and *z*_0_ (m) is the distance from the surface of the TO to the center of the excitation coil.

For a stationary sample consisting of a homogeneous ferromagnetic sheet material, Equations (3), (5) and (6) reduce to the following form:

(8) $$\kappa =\frac{\gamma -\varsigma \mu_{r}}{\gamma +\varsigma \mu_{r}}$$

where

(9) $$\gamma =\sqrt{\xi^{2}+\eta^{2}+j\omega \sigma \mu_{0}\mu_{r}}$$

where *σ* (S/m) is the electrical conductivity of the homogeneous material, and *µ_r_* is the relative magnetic permeability of the homogeneous material.

Software for calculating the SECP output signal was developed on the basis of the magnetodynamic model in Equation (1), with Equations (8) and (9) taken into account. The double improper integrals are evaluated numerically using the Gauss–Kronrod quadrature method with a relative error of 1·10^−3^. The software was verified and validated by comparing its results with those of the physical experiments and finite element method simulations reported in [30]. The same test case was used in the validation calculations [24]. Therefore, the numerical results obtained using this software are reliable within the range of validity of the adopted assumptions. 

### 2.2. Taguchi-Based Robust Parametric Design of Eddy Current Profile Gauges

Taguchi’s robust parameter design method was developed for quality control engineering. The method is intended to reduce variability in the performance characteristics of processes or products as early as the design stage [31,32,33,34,35]. During the design process, the analysis focuses on a target performance metric that mathematically combines the mean and variance of the response. Taguchi defined this metric as the signal-to-noise ratio (*SNR*). In this context, noise represents uncertainty caused by uncontrollable disturbance factors such as lift-off. The method is not described in detail here because it is well established and widely used in various fields. The authors have adapted this method for the robust design of eddy current probes used to measure various physical quantities, as described in detail in [24,25,26]. These studies describe its application to this class of design problems. The design procedure seeks to maximize the achievable signal-to-noise ratio. The target response is evaluated at selected discrete levels of the controllable and uncontrollable parameters. Formally, this procedure is implemented through a design of experiments based on orthogonal arrays with the required properties. Once the simulation data obtained from the model in Equation (1), as modified by Equations (8) and (9), have been organized according to the experimental design, the signal-to-noise ratio is calculated using the following expression, which incorporates the quality-loss function (*Fitness*):

(10) $$SNR=-10\;\log_{10}\left(Fitness\right)$$

where

(11) $$Fitness=\frac{1}{n}\;\sum_{i=1}^{n}\frac{1}{{\left(\mathrm{mod}\left(E_{i}\right)\right)}^{2}}$$

where *E_i_* is the probe output signal, normalized in advance to a reference value of 1 V, and *n* is the sample size.

The *Fitness* function is inversely proportional to the squared output signal, thereby promoting maximization of the useful probe-signal level. A decrease in the value of *Fitness* corresponds to an increase in the signal amplitude. In this study, the loss function in Equation (10) was selected to satisfy Taguchi’s “larger is better” criterion. To determine the Taguchi optimal gauge parameters, the mean *SNR* is calculated for each parameter at each factor level. The optimal parameter combination is determined by selecting, for each parameter, the factor level associated with the maximum mean *SNR*. 

Several characteristics of profile gauges used to measure the properties of magnetic sheet materials should be emphasized. The uncontrollable factors that cause variation in the SECP output signal and whose effects must be minimized include lift-off and local variations in sheet thickness. Because several material grades are considered, the electrophysical property values used in the *SNR* calculations should be averaged across all relevant grades.

After determining the SECP parameters that maximize the signal-to-noise ratio while minimizing output signal variability caused by uncontrollable disturbance factors, a confirmatory numerical experiment must be conducted using the identified parameter combination to verify the effectiveness of the resulting design. The design is confirmed if its *SNR* exceeds the *SNR* values obtained for all configurations included in the design of experiments. The robustness of an SECP with optimal design parameters should be verified through Monte Carlo numerical experiments in which disturbances affecting the EMF measurements are repeatedly simulated, both individually and in combinations occurring simultaneously. Performance should be evaluated by comparing the output signal variability in the optimized and nonoptimized SECP designs and by calculating the sum of relative errors (*SRE*). The statistical significance of the effects of the probe parameters on the *SNR* is evaluated using analysis of variance (ANOVA) and Fisher’s test [36,37,38,39,40].

## 3. Results

### 3.1. Modelling Results

To illustrate the proposed approach, we first identify the material grades to be sorted that have similar electrophysical properties. The selected materials and their properties are presented in Table 1 [41,42].

For the subsequent calculations aimed at developing an optimal SECP design capable of effectively suppressing the effects of uncontrollable factors on the signal, we used the class-averaged electrophysical properties of the specified materials. Although this assumption is not universally applicable, it is appropriate for developing a single probe design capable of classifying all the specified material grades. This approach differs fundamentally from the authors’ previous applications of the method.

Constructing a Taguchi design of experiments involves selecting orthogonal arrays according to the numbers of controllable and uncontrollable factors. The controllable SECP parameters and their variation ranges are presented in Table 2, whereas the uncontrollable factors and their variation ranges are presented in Table 3.

Thus, the design of experiments includes nine controllable parameters and two uncontrollable noise parameters. All these parameters are subsequently evaluated at five gradation levels. Based on the numbers of factors and gradation levels, the standard orthogonal array templates L_50_(2^1^,5^11^) and L_25_(5^6^) were selected [43,44,45]. The first array, L_50_(2^1^,5^11^), comprises 50 experimental runs, with one factor evaluated at two levels and the remaining factors evaluated at five levels. The study employed a modified L_50_(5^9^) orthogonal array that accommodates nine controllable factors. This array was derived from the standard L_50_(2^1^, 5^11^) template by removing the column corresponding to the two-level factor and two redundant columns corresponding to five-level factors. This modification is permissible because removing these columns preserves the orthogonality of the array. The second orthogonal array comprises 25 experimental runs representing combinations of six uncontrollable factors, each evaluated at five levels. Because only two uncontrollable factors are considered in the present study, this array was modified to the form L_25_(5^2^). 

The Taguchi design of experiments procedure involves calculating *SNR* values from the computer simulation data using Equation (10). Table 4 presents the design of experiments values of the controllable parameters in physical units, and Table 5 presents the corresponding values of the noise factors.

In accordance with the Taguchi design of experiments methodology, the EMF was calculated for each of the 50 probe design variants listed in Table 4 under the 25 combinations of uncontrollable noise factors listed in Table 5. Selected numerical results are presented in Table 6. These values were calculated using the mathematical model in Equation (1) together with Equations (8) and (9), with an excitation current of *I* = 0.3 A, *N*_1_ = 180 turns in the excitation coil, and *N*_2_ = 200 turns in the pick-up coil. In the modeling, the TO was represented as a sheet material with averaged electrophysical property values of *µ* = 170 and *σ* = 5.88 MS/m.

**Figure 2:**
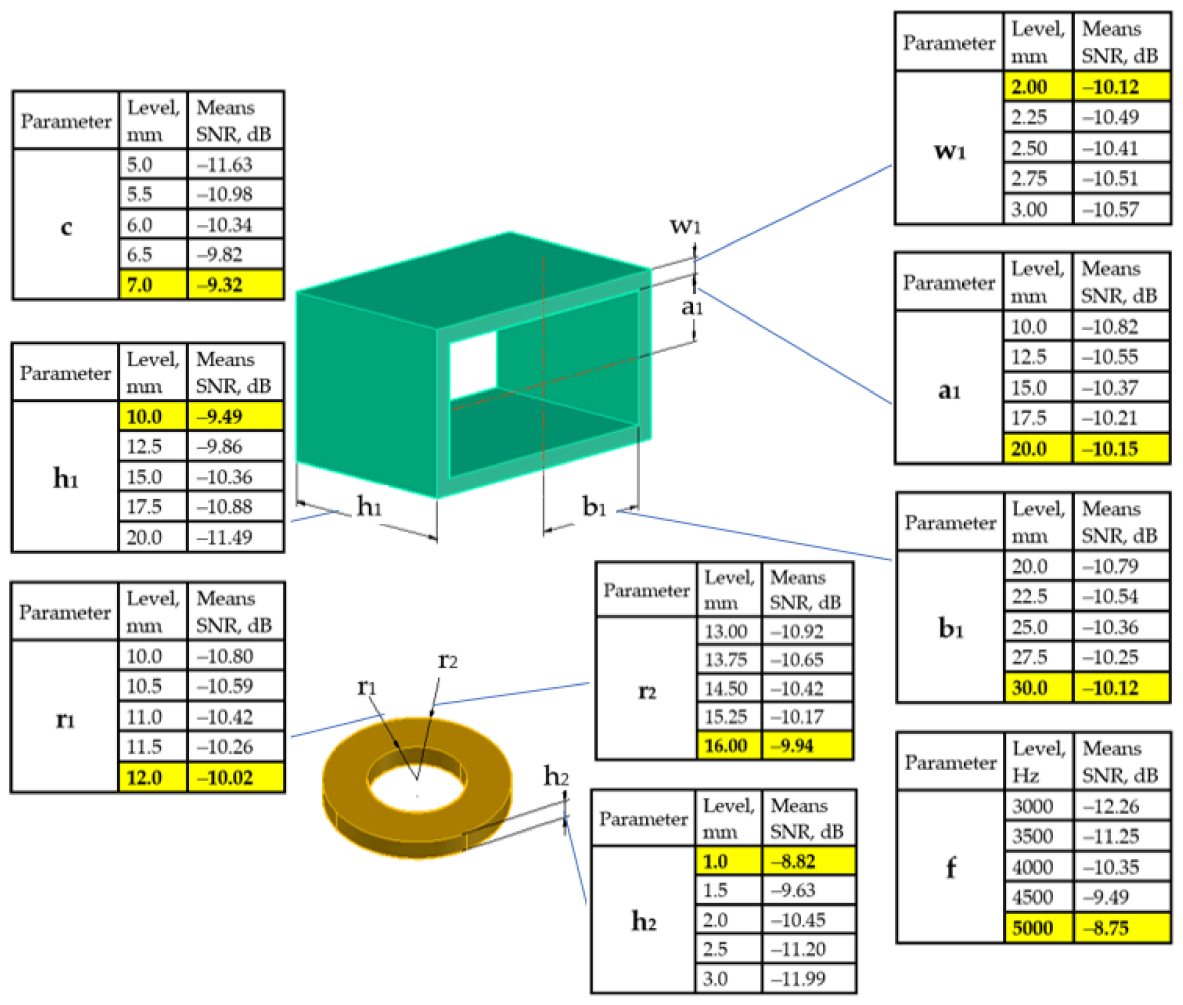
Procedure for selecting the optimal profile gauge parameters.

Based on the calculated EMF values, the signal-to-noise ratio was determined for each planned experiment; selected results are presented in Table 7. To determine the probe parameters that provide the highest possible *SNR*, the *SNR* values were grouped by parameter and factor level, and the mean *SNR* was calculated for each group. The optimal probe parameter set and operating mode were selected by identifying the maximum mean *SNR* for each parameter at the corresponding factor level, as highlighted in Figure 2. The final set of Taguchi-optimized parameters for the SECP design is presented in Table 8.

### 3.2. Confirmatory Numerical Experiment for Establishing the Optimal Design of a Profile Gauge

**Figure 3:**
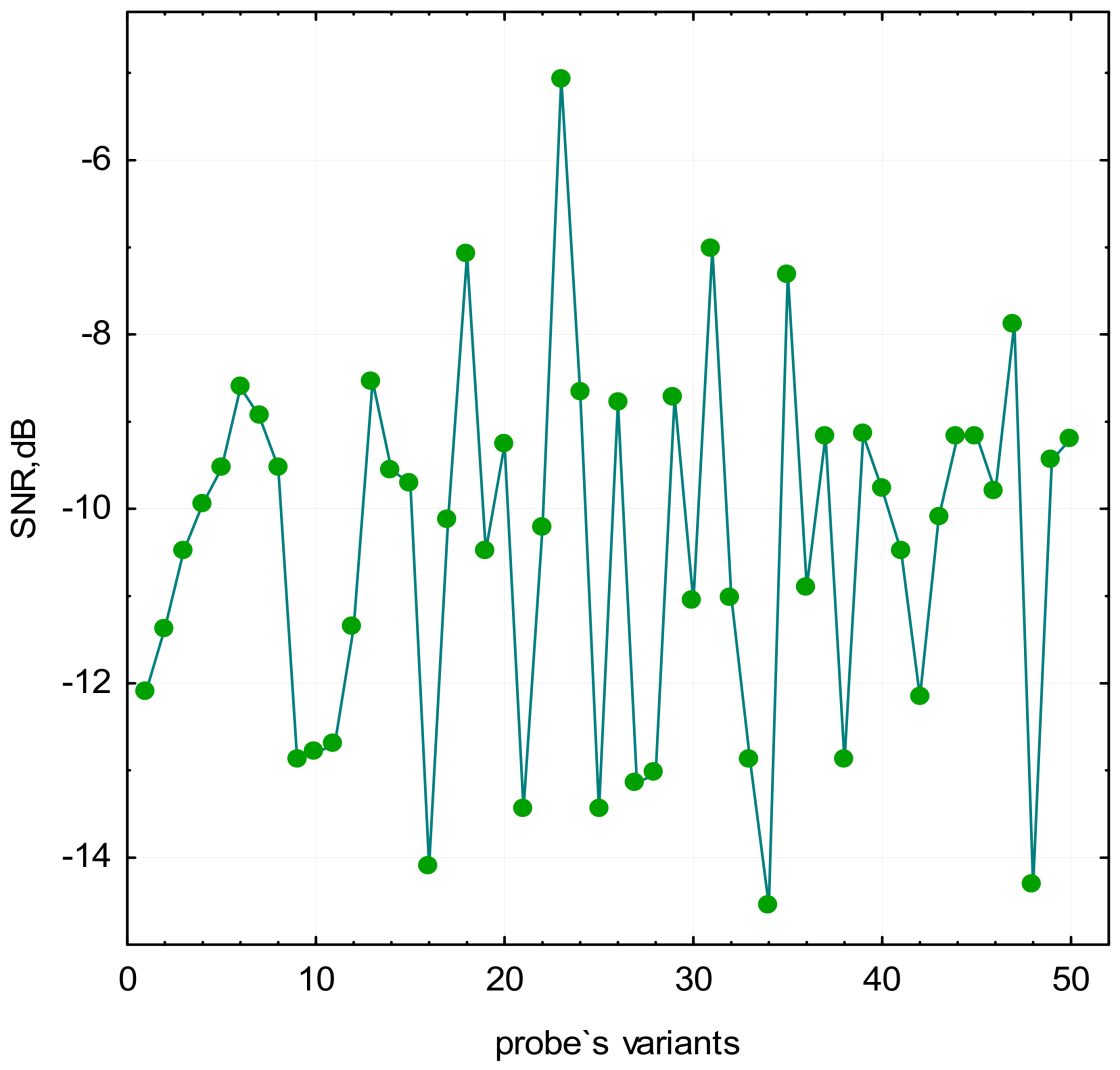
*SNR* values for all analyzed SECP design variants.

A confirmatory numerical experiment was conducted to determine the signal-to-noise ratio achieved with the optimal SECP design. The same sets of uncontrollable noise parameters were used in this experiment, and the corresponding EMF and *SNR* values were determined. The optimal design with the parameters listed in Table 8 produced a signal-to-noise ratio of −3.42 dB. In contrast, the nonoptimized design variants produced lower *SNR* values. Selected values are presented in Table 7, and the results for all 50 designs are shown in Figure 3.

Consequently, the optimal design provides a higher signal-to-noise ratio than any of the nonoptimized variants.

### 3.3. Numerical Study of Probe Signal Variability Using the Monte Carlo Method

Monte Carlo numerical simulations were conducted to evaluate the effectiveness of reducing the effects of noise factors that cause the probe output signal to deviate from its undisturbed value. The optimal SECP parameters were used to calculate the output signal under the influence of the noise parameters lift-off *z*_1_ and sheet-material thickness *d*. Thirty simulation realizations were generated by randomly sampling these uncontrollable noise parameters from uniform distributions over the ranges specified in Table 3. For comparison, the same analysis was performed for the best nonoptimal design, No. 23, and the worst nonoptimal design, No. 34, selected from Table 4.

Table 9 presents the numerical values of the noise factor *z*_1_ and the corresponding SECP output signals for the optimal design, *E_opt_*; the best nonoptimal design, *E_best_*; and the worst nonoptimal design, *E_worst_*. Table 9 also presents the relative EMF deviations calculated for each simulation. These deviations quantify the difference between the noise-affected and undisturbed signals and are calculated as follows:

(12) $$\delta_{i}=\frac{\left|E_{i}-E_{undis}\right|}{E_{undis}}$$

where *E_i_* (V) is the output signal of the corresponding probe in the *i*-th Monte Carlo simulation (*i* = 1, …, 30), and *E_undis_* (V) is the undisturbed probe signal value.

To compare the effectiveness of the SECP designs in suppressing the effects of uncontrollable noise factors, the sum of relative errors (*SRE*) was calculated as follows:

(13) $$SRE=\sum_{i=1}^{30}\left|\delta_{i}\right|$$

A similar study was conducted in which the local TO thickness was treated as a noise factor and varied from 4.7 to 5.3 mm. The numerical-modeling results showed no significant effect of this factor on the SECP output signal, regardless of the probe design. Therefore, the relative deviations *δ*, which were on the order of 10^−11^, were considered negligible.

### 3.4. Analysis of Variance

To determine whether the controllable factors had significant effects on the *SNR*, ANOVA was performed using Fisher’s test as described in [36,37,38,39,40,41]. The applicability of ANOVA was evaluated by testing whether the experimental data were normally distributed. For this purpose, the D’Agostino–Pearson test, which combines tests of skewness and kurtosis, was used [43]. Table 10 presents selected results of this test, including the DA statistic and the minimum and maximum *p*-values. Because the obtained *p*-values exceeded the significance level of 0.05, the null hypothesis of normality was not rejected, supporting the use of ANOVA.

The mean square (*MS*) was calculated for each design parameter, together with the residual mean square (*MS_residual_*), and the corresponding ratios were evaluated. The significance of each parameter effect was evaluated using the following condition:

(14) $$\frac{MS}{MS_{residual}}>F_{\alpha ,k_{1},k_{2}}$$

where α is the significance level, set to 0.05, $F_{\alpha ,k_{1},k_{2}}$ is the tabulated critical value of Fisher’s *F*-distribution for the specified α, *k*_1_ is the number of degrees of freedom associated with the corresponding factor, and *k*_2_ is the number of residual degrees of freedom.

If Condition (14) is satisfied, the effect of the factor is considered statistically significant; otherwise, it is considered nonsignificant. Table 11 presents the ANOVA results obtained using the L_50_(5^9^) orthogonal array for the controllable factors and the external L_25_(5^2^) orthogonal array for the noise factors. The number of degrees of freedom for each factor was calculated as *k*_1_ = *a*−1, where *a* is the number of levels of the corresponding factor. The residual degrees of freedom were calculated as $k_{2}=N-1-\sum_{i=1}^{9}k_{1i}$, where *N* = 50 is the total number of experiments in the design. Table 11 uses the following notation: *SS* is the sum of squares for the factor or residual component, *F* is the variance ratio, *p* is the *p*-value, and *Q* is the percentage contribution of each factor to the total variation. In deterministic numerical modeling, residual variation arises not from random measurement error, as it does in a physical experiment, but from unmodeled factor interactions, nonlinearities, and numerical errors. The *F*-ratios and *p*-values are calculated relative to the residual variance *MS_residual_*. These statistics indicate whether the main effect of a given design parameter exceeds the variation attributable to unmodeled interactions and residual effects within the selected experimental design.

The table value of Fisher’s criterion for this study is $F_{0.05,4,13}=3.18$. A comparison of the actual variance ratios obtained for each factor shows that all parameters are statistically significant according to Fisher’s criterion. Secondary factors were not combined, since all primary design parameters showed a statistically significant effect. The statistical indicators of the ANOVA were used to rank the influence of design parameters on the *SNR*.

## 4. Discussion

Thus, an optimal probe design has been identified for sorting samples of sheet magnetic material, providing the maximum possible signal-to-noise ratio, −3.42 dB, which is higher than any values of this ratio for nonoptimized variations of the SECP designs. Thus, the maximum *SNR* value is *SNR*_max_ = −5.06 dB, which corresponds to the best nonoptimized design, number 23, while the minimum value of this ratio is *SNR*_min_ = −14.55 dB, which corresponds to the worst design, number 34. In other words, under the specified boundary conditions, the maximum increase in the signal-to-noise ratio will be 11.13 dB, and the minimum will be 1.64 dB. Even with a minimal *SNR* increase, the probe signal level was found to grow by approximately 15% relative to the mean for the optimal design, demonstrating the significant practical significance of the proposed design method.

A series of numerical experiments based on the Monte Carlo statistical modeling method has demonstrated that, as the *SNR* increases, the variations in the output signals of the optimal probe design decrease in both cases when various noise factors are applied. Thus, when varying the lift-off *z*_1_, the *SRE* value is 4.33 for the optimal SECP design, 4.37 for the best nonoptimal design, and 4.5 for the worst nonoptimal design. Therefore, the optimal design achieves the best value for this parameter. During the research, it was found that local variations in sheet thickness had almost no effect on the output signal of the SECP.

As a result of ANOVA, the priority rankings of the influence of the following parameters on the *SNR* of the profile gauge were established: excitation frequency, the height of the pick-up coil’s cross-section, the center-to-center distance between the pick-up and excitation coils, the height of the excitation coil cross-section, the outer and inner radii of the pick-up coil, the width and length of the excitation coil, and the width of the excitation coil cross-section.

## 5. Conclusions

The article presents a new concept for the eddy current classification of ferromagnetic sheet materials by grades in difficult cases where the profiles of their material properties are similar; this concept is based on a proposed methodology for using inversion features in a lower-dimensional latent space. 

In this study, as the first stage of the concept, the method for improving the *SNR* on the profiles gauge of material properties TO, designed for the fine-grade classification of sheet magnetic materials, has been generalized and adapted, while simultaneously suppressing uncontrollable noise factors based on robust parametric design of their structures using Taguchi’s optimization algorithm. 

A confirmatory numerical experiment has shown that the optimal design of the SECP discovered in this study ensures the maximum signal-to-noise ratio, which is higher compared to any values of this ratio for their nonoptimized variants.

Numerical experiments using the Monte Carlo statistical modeling method have demonstrated that the optimal design of the SECP identified in this study yields the lowest *SRE* value for the signal when subjected to noise factors. An ANOVA was performed to determine the ranking of the effects of design and operating parameters on the *SNR* of the profile gauge.

Future research will focus on the following stages of the proposed concept of eddy current classification of materials by grade, specifically the inverse determination of material property profiles using an informatively pure signal obtained directly from the probe, and the application of generative adversarial networks to generate realistic material property profiles, solving the classification problem in latent GAN space.

## Figures and Tables

**Table 1:** Carbon steel grades and their properties.

Properties	Grade				
	S355JR	C22E	C35E	C45E	C50E
*µ_r_*	200	250	150	100	150
*σ*, MS/m	6.7	6.9	6.3	5.8	3.7

**Table 2:** Controllable SECP parameters and their variation ranges.

Controllable Parameters	Variation Range
Width of the excitation coil *a*_1_, m	0.010–0.020
Length of the excitation coil *b*_1_, m	0.020–0.030
Height of the excitation coil cross-section *h*_1_, m	0.010–0.020
Width of the excitation coil cross-section *w*_1_, m	0.002–0.003
Inner radius of the pick-up coil *r*_1_, m	0.010–0.012
Outer radius of the pick-up coil *r*_2_, m	0.013–0.016
Height of the pick-up coil cross-section *h*_2_, m	0.001–0.003
Center-to-center distance between the pick-up and excitation coils *c*, m	0.005–0.007
Excitation frequency *f*, kHz	3–5

**Table 3:** Uncontrollable parameters and their variation ranges.

Uncontrollable Parameters	Variation Range
Lift-off *z*_1_, 10^−3^ m	1.5–3.0
Sheet material local thickness *d*, 10^−3^ m	4.7–5.3

**Table 4:** Design of experiments for the controllable parameters.

No.	Controllable Parameters								
	*a*_1_, mm	*b*_1_, mm	*h*_1_, mm	*w*_1_, mm	*r*_1_, mm	*r*_2_, mm	*h*_2_, mm	*c*, mm	*f*, kHz
1	10.0	20.0	10.0	2.00	10.0	13.00	1.0	5.0	3.0
2	10.0	22.5	12.5	2.25	10.5	13.75	1.5	5.5	3.5
3	10.0	25.0	15.0	2.50	11.0	14.50	2.0	6.0	4.0
4	10.0	27.5	17.5	2.75	11.5	15.25	2.5	6.5	4.5
5	10.0	30.0	20.0	3.00	12.0	16.00	3.0	7.0	5.0
…	…	…	…	…	…	…	…	…	…
20	17.5	30.0	15.0	2.00	11.5	13.75	2.5	5.5	5.0
21	20.0	20.0	20.0	2.75	11.0	13.75	2.5	6.0	3.5
22	20.0	22.5	10.0	3.00	11.5	14.50	3.0	6.5	4.0
23	20.0	25.0	12.5	2.00	12.0	15.25	1.0	7.0	4.5
24	20.0	27.5	15.0	2.25	10.0	16.00	1.5	5.0	5.0
…	…	…	…	…	…	…	…	…	…
32	12.5	22.5	15.0	2.25	11.5	15.25	2.0	7.0	3.0
33	12.5	25.0	17.5	2.50	12.0	16.00	2.5	5.0	3.5
34	12.5	27.5	20.0	2.75	10.0	13.00	3.0	5.5	4.0
…	…	…	…	…	…	…	…	…	…
46	20.0	20.0	20.0	2.25	10.5	16.00	2.0	6.5	4.5
47	20.0	22.5	10.0	2.50	11.0	13.00	2.5	7.0	5.0
48	20.0	25.0	12.5	2.75	11.5	13.75	3.0	5.0	3.0
49	20.0	27.5	15.0	3.00	12.0	14.50	1.0	5.5	3.5
50	20.0	30.0	17.5	2.00	10.0	15.25	1.5	6.0	4.0

**Table 5:** Design of experiments for the interference parameters.

No.	Parameter Values												
	1	2	…	9	10	11	12	…	17	18	…	24	25
*z*_1_, mm	1.50	1.50	…	1.87	1.87	2.25	2.25	…	2.62	2.62	…	3.00	3.00
*d*, mm	4.70	4.85	…	5.15	5.30	4.70	4.85	…	4.85	5.00	…	5.15	5.30

**Table 6:** EMF values obtained for the analyzed SECP designs.

No.	Noise Cortege, ×10^−3^ m	EMF, mV							
		1	2	…	34	35	…	49	50
1	{1.5;4.7}	317.18	337.58		226.97	536.60		411.91	421.71
…	…	…	…	…	…	…	…	…	…
7	{1.875;4.850}	283.55	304.55		207.53	484.97		375.32	385.13
…	…	…	…	…	…	…	…	…	…
13	{2.25;5.00}	253.93	275.15		189.97	438.98		342.39	352.15
…	…	…	…	…	…	…	…	…	…
19	{2.625;5.150}	227.78	248.95		174.09	397.95		312.74	322.39
…	…	…	…	…	…	…	…	…	…
25	{3.0;5.3}	204.66	225.57		159.73	361.30		285.99	295.50

**Table 7:** Signal-to-noise ratio values for the SECP designs.

Experiment Number	1	2	3	…	22	23	…	33	34	…	48	49	50
*SNR*, dB	−12.09	−11.37	−10.48	…	−10.22	−5.06	…	−12.88	−14.55	…	−14.32	−9.44	−9.19

**Table 8:** Optimal SECP design parameters.

Taguchi’s Parameters	*a*_1_, mm	*b*_1_, mm	*h*_1_, mm	*w*_1_, mm	*r*_1_, mm	*r*_2_, mm	*h*_2_, mm	*c*, mm	*f*, kHz
Values	20	30	10	2	12	16	1	7	5

**Table 9:** SECP output signals for variations in lift-off *z*_1_.

No.	*z*_1_, mm	*E_opt_*, mV	*δ_opt_*	*E_best_*, mV	*δ_best_*	*E_worst_*, mV	*δ_worst_*
1	1.571	787.802	0.0075	669.514	0.0056	220.000	0.0166
2	2.005	708.531	0.1073	599.979	0.1089	198.738	0.1116
3	2.098	692.756	0.1272	586.174	0.1294	194.492	0.1306
4	2.919	569.682	0.2823	481.487	0.2848	158.168	0.2930
5	2.301	659.699	0.1688	557.279	0.1723	184.576	0.1750
…	…	…	…	…	…	…	…
15	2.365	649.655	0.1815	550.204	0.1827	182.862	0.1826
16	1.833	738.803	0.0692	626.502	0.0695	206.873	0.0753
17	1.506	800.526	0.0085	679.055	0.0086	221.800	0.0086
18	1.623	777.789	0.0201	658.716	0.0216	217.322	0.0286
19	2.783	588.212	0.2589	497.614	0.2609	165.008	0.2624
20	2.717	597.454	0.2473	505.663	0.2489	166.719	0.2548
…	…	…	…	…	…	…	…
26	2.467	634.007	0.2012	537.541	0.2016	168.629	0.2462
27	1.628	776.834	0.0213	659.877	0.0198	217.066	0.0297
28	2.922	569.281	0.2827	481.138	0.2854	158.059	0.2935
29	1.912	724.719	0.0869	614.157	0.0878	203.091	0.0922
30	2.973	562.511	0.2913	475.251	0.2941	157.215	0.2972

**Table 10:** Results of the D’Agostino–Pearson test for data normality.

Experiment Number	1	…	13	14	…	19	20	…	35	…	50
DA-stat	5.436	…	5.771	5.523	…	5.507	5.114	…	5.492	…	5.551
*p*-value	0.066	…	0.055	0.063	…	0.064	0.077	…	0.064	…	0.062

**Table 11:** Results of the ANOVA.

Parameters	*SS*	*k*	*MS*	*F*	*p*-Value	*Q (*%)
*a*_1_	2.9311	4	0.7328	40.04	0.344·10^−6^	1.36
*b*_1_	2.6758	4	0.6689	36.55	0.589·10^−6^	1.24
*h*_1_	25.3667	4	6.3417	346.54	0.461·10^−12^	11.77
*w*_1_	1.2479	4	0.3119	17.04	0.435·10^−4^	0.58
*r*_1_	3.6020	4	0.9005	49.21	0.100·10^−6^	1.67
*r*_2_	6.0132	4	1.5033	82.15	0.429·10^−8^	2.79
*h*_2_	62.8326	4	15.7082	858.37	1.321·10^−15^	29.15
*c*	33.5537	4	8.3884	458.38	7.607·10^−14^	15.57
*f*	77.0609	4	19.2652	1052.74	3.522·10^−16^	35.75
*Residual*	0.2379	13	0.0183			0.11
*Total*	215.5218	49				100

## Data Availability

Dataset available on request from the authors.

## Abbreviations

MECT	Multi-frequency eddy current testing
PECT	Pulsed eddy current testing
MIS	Magnetic induction spectroscopy
PCA	Principal component analysis
GAN	Generative adversarial network
TO	Tested object
SECP	Surface eddy current probe
EMF	Electromotive force
SNR	Signal-to-noise ratio
SRE	Sum relative error
ANOVA	Analysis of Variance
